# Efficacy of front-of-pack warning label system versus guideline for daily amount on healthfulness perception, purchase intention and objective understanding of nutrient content of food products in Guatemala: a cross-over cluster randomized controlled experiment

**DOI:** 10.1186/s13690-023-01124-0

**Published:** 2023-06-16

**Authors:** María Fernanda Kroker-Lobos, Analí Morales-Juárez, Wilton Pérez, Tomo Kanda, Fabio S Gomes, Manuel Ramírez-Zea, Carolina Siu-Bermúdez

**Affiliations:** 1 INCAP Research Center for the Prevention of Chronic Diseases, Institute of Nutrition of Central America and Panama, Calzada Roosevelt 6-25 zona 11, Guatemala City, Guatemala; 2 INCAP Unit Planning, Institute of Nutrition of Central America and Panama, Calzada Roosevelt 6-25 zona 11, Guatemala City, Guatemala; 3 Pan American Health Organization/World Health Organization, Diagonal 6 10-50 zona 10, Guatemala City, Guatemala; 4 Pan American Health Organization/World Health Organization, 525 23rd St NW, 20037 Washington, DC USA

**Keywords:** Food labelling, Warning label system, GDA, Guatemala

## Abstract

**Background:**

Front-of-package warning labels (FOPWL) have been adopted in many countries aiming at reducing the consumption of unhealthy food and drink products and have also been considered in Guatemala. The aim of the study is to evaluate the efficacy of FOPWL versus Guidelines for Daily Amount (GDA) on products’ healthfulness perception (HP), purchase intention (PI) and the objective understanding of the nutrient content (UNC) in Guatemala.

**Methods:**

Participants (children and adults) (n = 356) were randomly assigned to evaluate either FOPWL or GDA during a crossover cluster randomized experiment in rural and urban areas across 3 phases of exposure. During phase 1, participants evaluated mock-up images of single products (single task) and compared pairs of products within the same food category (comparison task) without any label. In phase 2, participants evaluated labels only (without any product), and during phase 3, they evaluated the same products and questions from phase 1, now depicting the assigned front-of-package label. We generated indicators for single-task questions and scores for comparison tasks, one for each HP, PI and UNC questions. We used intention-to-treat, difference-in-difference regression analysis to test whether exposure to FOPWL was associated with HP, PI and UNC, compared to GDA. We also tested models for children and adults and by area (rural/urban) separately adjusting for sociodemographic variables.

**Results:**

In single tasks, FOPWL significantly decreased the PI (β -18.1, 95%CI -23.3, -12.8; p < 0.001) and the HP (β -13.2, 95%CI -18.4, -7.9; p < 0.001) of unhealthy food products compared to GDA. In the comparison task, FOPWL significantly increased the UNC (β 20.4, 95%CI 17.0, 23.9; p < 0.001), improved PI towards healthier choices (OR 4.5, 95%CI 2.9, 7.0 p < 0.001) and HP (OR 5.6, 95%CI 2.8, 11.1; p < 0.001) compared to GDA. Similar results were found in children and adults and in urban and rural settings.

**Conclusions:**

FOPWL reduces products’ healthfulness perception and purchase intention, and increases understanding of products’ nutrient content compared to GDA.

**Supplementary Information:**

The online version contains supplementary material available at 10.1186/s13690-023-01124-0.

**Table:** 

**Text box 1. Contributions to the literature**
Previous research has shown the effectiveness of front-of-pack warning labels (FOPWL) to select healthier foods in countries with high levels of urbanization. However, little is known of its impact in Central American countries with an important proportion of rural populations and with lower levels of education.
Findings from this study, carried out in Guatemala, showed that FOPWL is an effective tool to select healthier choices in adults and children, from rural and urban communities and with less than 6 years of education.
These findings contribute to promote the adoption of the FOPWL system in Guatemala and Central American countries.

## Background

Non-communicable diseases (NCDs) are the leading causes of disability and premature mortality in the Central American region, where more than 50% of adults have overweight or obesity [1, 2]. The consumption of processed and ultra-processed food products with excessive amounts of energy and critical nutrients (such as total fats, saturated fats, trans fats, sodium and added sugars) is associated with increased risk of overweight and obesity; NCDs such as diabetes, cardiovascular diseases, cerebrovascular diseases and cancers; depression; and all-cause mortality in adults [3–10]. Additionally, these products also worsen the diets and health of children and adolescents by displacing breastfeeding, fruits and vegetables, increasing saturated fats, sugars and sodium intake, blood lipid levels and body fat [11–15]. Childhood obesity is a direct cause of gastrointestinal, musculoskeletal and orthopedic complications, sleep apnea, and the accelerated onset of cardiovascular disease and type-2 diabetes [16].

Front-of-pack warning labeling (FOPWL) has been recognized as an effective policy tool for the prevention of NCDs in the Americas according with the Pan American Health Organization (PAHO/WHO) [17]. There is growing evidence demonstrating that FOPWL systems – which allow consumers to quickly, easily and correctly identify products that are excessive in critical nutrients – are an effective health policy tool to improve consumers’ understanding, perception and purchase decisions, helping to tackle unhealthy diets and promote healthier food environments. [17–22].

Argentina, Brazil, Canada, Chile, Colombia, México, Peru, Uruguay and Venezuela have adopted octagonal warning labels [23]. In Chile, said FOPWL system has proven to reduce purchases of sugar-sweetened beverages and breakfast cereals [22]. In Uruguay, it also demonstrated to immediately increase the consumers’ ability to identify products with excessive content of sugar, fat, saturated fat and sodium [21], and have been projected to prevent 1.3 million cases of obesity in Mexico over five years [20]. Moreover, recent evidence from Peru demonstrated that food products without warning labels decreased from 16 to 5%, 36 months after implementation of the policy [24].

On the other hand, a mandatory based-evidence front-of-pack food labelling in Central America is absent. Previous research in Guatemala has found deficiencies in the declaration of nutrients related to NCDs in the form of nutrition facts tables. For example, food labeling technical regulations are not mandatory and an important proportion of processed and ultraprocessed products do not declare total sugars and trans fats. Additionally, most processed and ultra-processed products in Guatemalan supermarkets are excessive in critical nutrients [25].

In 2015, the “Strategy for the prevention of overweight and obesity in childhood and adolescence 2014–2025” [26], approved by the Council of Ministries of Health of Central America and Dominican Republic (COMISCA), recommended the implementation of a FOPWL on packaged food and beverage products, including the regulation of marketing to children and adolescents. In 2017, COMISCA submitted to the Central American Secretariat for Economic Integration (SIECA) a proposal to update the Central American Food Labelling Technical Regulation (RTCA) with the inclusion of a FOPWL system that consisted of octagonal warning labels [27]. A counterproposal was formulated and submitted to SIECA by the economy sector in response, suggesting the adoption of the Guideline Daily Amounts (GDA) labeling in spite of the robust body of evidence that has demonstrated the ineffectiveness of that system [19]. Similarly, a recent law initiative in Guatemala (Bill 5504 “Promotion of Healthy Eating”) called for a FOPWL and the regulation of food marketing to children in 2018. However, no progress has been achieved by Congress so far regarding the initiative [28].

Label understanding is a key feature of label use and effectiveness [29]. According to PAHO/WHO, the aim of a regulatory FOPWL system is allowing consumers to correctly identify products with excessive amount of sugars, total fats, saturated fats, trans fats and sodium [17]. Hence, from a public health perspective, it is important to measure performance features that should include: understanding of the nutritional content, use of the information that impact food consumption (healthfulness perception), and consumer’s purchase decisions [17, 30]. For example, randomized trials in Brazil and Mexico have evaluated understanding of nutritional content and healthfulness perception and have found that FOPWL lead consumers to perceive ultraprocessed food products as less healthy and provide better understanding of the nutritional content compared with similar food products labeled with the traffic light system [31] and GDA [32]. In Mexico and Uruguay, randomized control trials have evaluated similar outcomes using online shopping simulations, and have shown that FOPWL guided consumers towards healthier choices compared with GDAs [33, 34]. More recently, a randomized experiment in Mexico, demonstrated that FOPWL helped to choose healthier options in children [35].However, countries that have evaluated or implemented the FOPWL system have higher levels of urbanization, higher education levels, and lower proportions (or absence) of indigenous populations. To date, the efficacy of the FOPWL has not been evaluated in Central American countries with a significant share of rural and indigenous populations and lower literacy (For example, 46% of the Guatemalan population lives in rural areas and illiteracy can reach one third of indigenous adults) [36]. More research is needed to understand whether there is a differential effect of warnings labels by education and by area of residence. On the other hand, evidence in children has been scarce [18]. Therefore, the purpose of this study is to examine the efficacy of FOPWL and GDA (both systems under consideration by Central American authorities); in the perceived healthfulness, purchase intention and objective understanding of the nutritional content of food products among children and adults from urban and rural areas of Guatemala.

## Methods

### Study design

A crossover cluster randomized controlled experiment was designed to evaluate the effect of two different front-of-package label (FOPL) systems over the participants’ perception of healthfulness, purchase intention and objective understanding of the nutrient content of food products. The crossover design used the participants as their own control, to attribute to the intervention any difference in the evaluated outcomes.

**Fig. 1:**
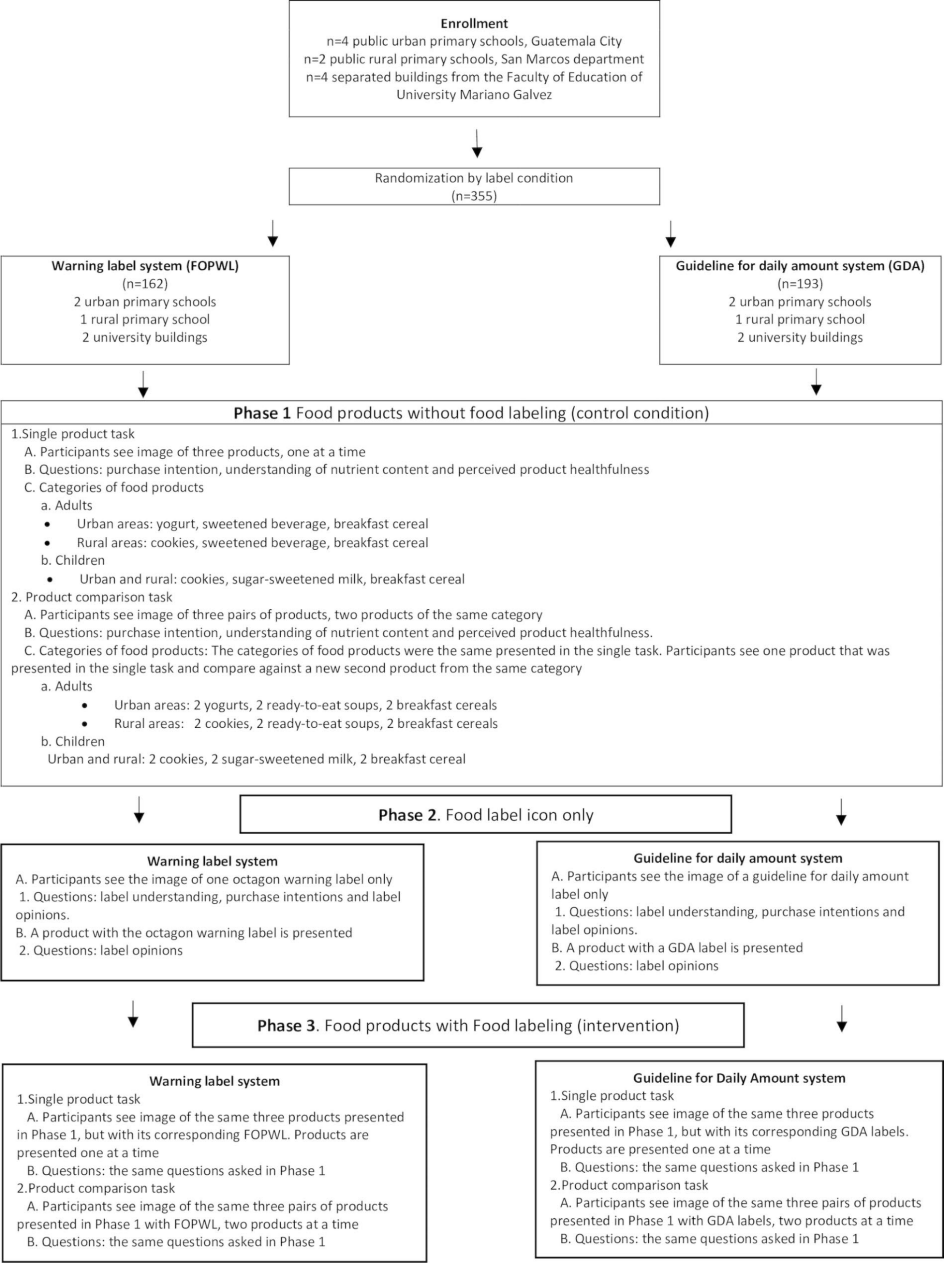
Flowchart of study and description of phases. The food products and questions were presented following the same sequence and a non-random order. Full list of questions is presented in Additional Table 1

Each participant was randomly assigned to one of the two arms of the study, FOPWL or GDA, and each participant acted as their own control. At phase 1 (control condition), participants were asked about their purchase intention, objective understanding of the nutritional content, and healthfulness perception of three food products from different categories, which were individually presented without any FOPL system (see Fig. 1). After answering about each product individually, participants were asked about the same indicators (i.e. purchase intention, objective understanding of the nutritional content, and product healthfulness perception) for three sets of two different products, each pair falling into one of the three categories. During phase 2, participants were presented solely with one of the FOPL system icons to increase familiarity with them. Participants were randomly assigned to see the labels only (i.e. octagonal warning label or GDA) without being attached to any product and were asked questions about their general perception and understanding about the icon. Lastly, at phase 3 (the intervention condition), participants were asked the same set of questions as during phase 1, but now the FOPL system was applied to the images of the same mock-up products shown to each participant in phase 1.

### Study sample

Four urban and two rural public primary schools were selected, in Guatemala City and in the department of San Marcos (located 250 km away from Guatemala City in the western highlands) respectively. Schools were selected by convenience. Schools were randomly assigned to evaluate either the FOPWL or the GDA. Upon agreement with school authorities, researchers organized meetings with children (8–12 years of age) and their mothers to invite them to participate in the study. Researchers provided detailed information of the study and provided informed consent to those interested in participating. Similarly, researchers approached university authorities from the Faculty of Education of University Mariano Galvez to request permission to recruit adults from four different buildings located in three different campuses (main campus in Guatemala City ant two satellite campus, one in Villa Nueva and the other in Boca del Monte). They were also randomly assigned to evaluate octagonal warning labels or GDA. To ensure different levels of education in the sample of adults, we recruited college students and faculty, and technical, administrative and maintenance staff from each building. Upon agreement to participate, researchers also provided detailed information of the study. The exclusion criteria (for the adult sample) were illiteracy, having a chronic condition (i.e. diabetes, hypertension), pregnancy and people with special diets, which could influence food choices (i.e., vegetarian, vegan, gluten free diets).

All participants provided written consent to participate in this study. In the case of children, parents or guardians provided a written consent for their child participation. Additionally, an oral assent was given by the child. The study was approved by the Ethics Committee of the Institute of Nutrition of Central America and Panama (CIE-REV 89/2019).

### Sample size

A sample size of 160 schoolchildren and 160 adults was estimated, assuming a power of 80% and significance at 0.05. Studies to examine the efficacy of FOPWL and GDA were not available in our study setting. Therefore, we assumed a moderate effect size (two-tailed) of 0.5 (standardized mean difference), as suggested by Cohen in the evaluation of purchase intention mean score of food products between the octagonal warning label group (80 schoolchildren, 80 adults) and the GDA (80 schoolchildren, 80 adults) [37]. The response rate was considered at 75%. Sample size was calculated using G*Power 3.1.9.2 (http://www.gpower.hhu.de/en.html).

### Pilot study

Using a previous validated questionnaire, researchers translated questions from Portuguese into Spanish [31]. Researchers carried-out a pilot study with 31 voluntary participants, ten children (5 GDA and 5 FOPWL) and 21 adults (11 GDA and 10 FOWPL). Researchers evaluated language, terms, understanding of questions and difficulty level, and changes were made accordingly. For example, in children, changes included the use of faces representing each response of the Likert scales (from the saddest to the happiest) [38]. In addition, the results of the pilot study revealed the importance of presenting different products to children (for some categories) than those targeting adults, and to explain some terms that were not easily recognized by some children such as trans fats and artificial sweeteners. The Spanish questionnaire is available in additional file 1. Details of the original questionnaire are described elsewhere [31].

### Study procedures

Images of mock-up food products with similar characteristics compared to well-known brands in Guatemala were designed. The selection of the category of products was based on previous evidence about the misunderstanding of healthfulness of certain products, food composition and labelling practices in Guatemala [25, 31]. Thus, ultraprocessed products that are easily identified as unhealthy were avoided such as soda, salty snacks, candies, etc. Three categories of food products were used in the adult sample in urban areas: yogurts, sweetened beverages and breakfast cereals. In rural areas, yogurts were replaced with cookies to increase familiarity with food products. In rural and urban areas, children evaluated cookies, sugar-sweetened milk and breakfast cereals. Images of the mock-ups evaluated by participants and their nutritional content are available in additional file 2.

Trained staff interviewed each participant after obtaining consent. At control condition or phase 1, participants’ perception of product healthfulness, purchase intention and objective understanding of the nutritional content were assessed by showing them mock-ups with no FOPL system depicted using single and comparison tasks. In the single task, they were shown a sequence of three different products, one at the time, and each from one of the three categories of products aforementioned. Additionally, in the comparison task, participants were also shown three sets of two mock-up products. Each set featured two mock-ups products from the same category but with different simulated brands: two brands of yogurts (cookies in rural areas), two brands of ready-to-eat soups, and two brands of breakfast cereals. Children evaluated two brands of cookies, two sugar-sweetened milks and two breakfast cereals.

Secondly at phase 2, the FOPWL or GDA icon was shown to participants without any package to assess their general understanding and impressions about the icons.

Lastly, at intervention condition or phase 3, participants were asked to respond to the same set of questions made at control conditions and were shown the same sequence of single and pairs of mock-ups, now depicting the assigned intervention – FOPL system, FOPWL or GDA. Figure 1 shows the phases of the study and the sequences of questions asked to the participants.

### Labelling conditions

#### Front-of-package warning label system (FOPWL)

**Fig. 2:**
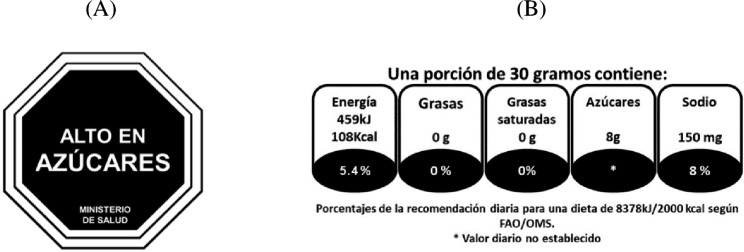
Examples of Front-of-package icons used. Octagonal nutritional warning label “High in Sugar” (FOPWL) (B) Guideline daily amount (GDA)

The same specifications (design, position and nutrients) proposed by COMISCA were used as one of the intervention conditions. This group was exposed to octagonal warning labels as exemplified by Fig. 2A, which is an FOPWL system that indicated when mock-up products were excessive in total sugars, fats, saturated fats, trans fats or sodium and contains artificial sweeteners. Octagonal black labels with a white contour contained the text “HIGH IN < name of the nutrient>” (“ALTO EN” in Spanish) with capital letters. The thresholds used to define if mock-up products were excessive in one or more critical nutrients were those found in the Pan American Health Organization (PAHO) nutrient profile model [39]. The octagons were always positioned at the upper right corner of the FOP.

### Guideline daily amounts (GDA) system

GDA labelling system’s icon, proposed by the food industry sector was used as the other intervention condition (Fig. 2B). The icon consists of a mono-chromatic miniature of the nutrition facts table and features the numeric amounts of energy (in calories and kilojoules), total fat (g), saturated fat (g), total sugar (g), and sodium(mg) for a given portion size of a food product, as well as the percentage of daily FAO/WHO recommendations of calories and nutrients these amounts represent assuming a diet fixed at 8738 Kilojoules/2000Kcal [40]. The icon depicts an asterisk (*) where the percentage of sugars would be placed, indicating that a daily recommendation has not been established. The GDA was positioned in the lower left corner of the FOP, to reflect the usual position applied by companies that use it voluntarily.

### Understanding of nutritional content

#### Understanding of nutritional content indicator

For single products, participants were asked to identify which critical nutrients were above recommended levels according to the PAHO nutrient profile [39]. Interviewers asked about each critical nutrient, one at a time (fats, total sugar, sodium, etc.). Interviewees could choose more than one option: total fat, total sugar, sodium, trans fat, saturated fat, artificial sweeteners and none. Correct responses were scored 1, and the total number was obtained by adding the correct responses and converting them into a 1-100 scale to create an understanding of nutritional content indicator.

### Understanding of nutritional content score

To evaluate two products of the same food group, participants were asked to compare and choose which product had a greater quantity for each critical nutrient. Interviewers asked about each critical nutrient, one at a time. Responses also included options such as “both have high levels”, “both have low levels” and “do not know/no response”. The PAHO nutrient profile was used to determine the correct responses in the comparison task [39]. Correct responses were scored 1, and the total number was obtained by adding the correct answers, and then converting them into a 1-100 scale to create a nutritional content score.

### Purchase intention

#### Purchase intention indicator

For single products, a 7-point Likert scale was used to evaluate the purchase intention, ranging from “1. I would definitely not buy it” to “7. I would definitely buy it” in response to the question: “Would you buy this product, or a similar one, for you or your family?”[41]. Likert scales were used as points (e.g. 1 = less likely to buy the product, 7 = more likely to buy the product) and the total number was obtained by adding the responses for each product and converting them into a 1-100 scale to create a purchase intention indicator (Additional file 1).

### Purchase intention score

To evaluate the purchase intention comparing two products from the same category, each participant was asked “Would you buy some of these products for you and your family?”. The question had a multiple-choice answer. To evaluate the change in purchase intention, correct responses were given a score of 1 if the person chose the healthier option. The total score was obtained by adding the number of correct answers to create a purchase intention score. The healthier option was the product with fewer octagons or those who responded “I would not buy neither of the two products” when necessary.

### Healthfulness perception

#### Healthfulness perception indicator

A 7-point Likert scale was used to evaluate product healthfulness perception, ranging from “1. Not healthy at all” to “7. Very healthy”, in response to the question: “Do you think this product is healthy?” [41]. Then, a perceived product healthfulness indicator created by using the Likert scale as points. The total number was obtained by adding the responses for each product and converting them into a 1-100 scale to create a healthfulness perception indicator (Additional file 1).

### Healthfulness perception score

To compare two products from the same food group, each participant was asked to choose the product they consider healthier from a multiple-choice question: “Could you indicate which product do you consider healthier?”. The PAHO nutrient profile was used to determine the correct responses in the comparison question [39]. The correct responses were scored 1 and total the number of correct answers was obtained by adding them.

### Co-variates

#### Socio-demographic characteristics

Sociodemographic characteristics such as sex, age, ethnicity, residency and education of participants were registered. Participants were coded as indigenous if they self-identified as indigenous and reported speaking an indigenous language. Residence was a dichotomous variable pre-defined by the study site as urban or rural. Education was measured as the total grades of schooling attained. For adults, we classified education defined as primary if the participant had attained six or more grades of schooling; or less than primary if participant had attained less than 6 grades of schooling.

### Analysis

#### Descriptive analysis

For children and adults, we summarized sociodemographic characteristics, such as age, sex, residency, ethnicity and education by label condition and phase of exposure. Similarly, we reported descriptive statistics for all outcomes by label condition and phase of exposure. Responses were converted into “indicators” for all single-task questions and into “scores” for comparison-task questions. Definitions of each score and indicators and how they were calculated are described in methods. Phase 1 or control condition represents the condition before any labelling was depicted. Phase 3 represents the intervention, i.e., the conditions after the corresponding label was presented to the participant. We also tested differences by label condition (FOPWL versus GDA) during phase 3 (the intervention exposure) using a T-test for all outcomes.

### Analysis of the effect of FOPWL on the nutritional content understanding, purchase intention and healthfulness perception compared to GDA

To assess the effect of FOPWL on the understanding of nutritional content, purchase intention and healthfulness perception, compared to GDA, we conducted difference-in-difference (DD) intention-to-treat analysis using fixed effects, generalized linear or ordinal regression models depending on the outcome’s distribution. We used ordinal regression models to estimate the odds of responding correctly more times such as in the case of healthfulness perception and purchase intention scores (comparison tasks). For the DD analysis, our primary interest was the interaction term between the phase of exposure (control or intervention) and label condition (FOPWL or GDA). This interaction term represents the differential effect of exposure to the FOPWL compared with GDA, after subtraction of the difference between the phases of exposure (Phase 1 versus Phase 3). The interaction term is, therefore, an estimate of the effect of FOPWL on the outcomes compared with GDA.

For each outcome, two models were tested. Model 1 (base model) included dummy variables for the phase of exposure and the label condition, as well as the previously described interaction term between phase of exposure and label condition, age and sex. In model 2, sociodemographic characteristics such as residency (rural/urban), ethnicity (indigenous/nonindigenous) and education (primary/less than primary) were added. For children we used education as a continuous variable. We tested models using pooled analysis (children and adults combined). Given the differences in cognitive development and products evaluated, we also tested separated models for children and adults. In pooled analysis, we also tested heterogeneity by residency and education through a third-order interaction term of phase of exposure, label condition and the corresponding variable (see Additional file 3). Standard errors were adjusted by cluster (school or university site). Two-sided significance was set as p < 0.05. All analyses were conducted in Stata 15.0 (College Station, Texas) and 95% confidence intervals (CI) were calculated.

## Results

### Sample characteristics

There were 355 participants, 162 in the FOPWL group and 193 in the GDA group, with nearly 50% of children in both groups (Table 1). Among children, there was more participation of females in the GDA group compared to the FOPWL group (59% vs. 41% p = 0.01). However, overall participation was above 60%, since recruitment focused on mothers of children attending the selected schools. More than 60% of participants resided in urban areas and recognized themselves as non-indigenous. On average, schooling was 5 years in children, and above 12 years in adults in both groups. More than 80% of adults have more than six grades of schooling and the proportion was even higher (94%) in the GDA group. No significant differences in age, residency and ethnicity were observed in both groups, for children and adults by label condition.

**Table 1:** Sociodemographic characteristics by label condition in children and adults, Guatemala, 2019

	Children (n = 177)			Adults (n = 178)		
	GDA (n = 92)	FOPWL (n = 85)	*p*	GDA (n = 101)	FOPWL (n = 77)	*p*
Age in years (mean ± SE)	10.7 ± 1.1	10.8 ± 1.1	0.241	34.9 ± 9.3	35.1 ± 9.6	0.855
Sex, female (%)	59	41	0.01	81	87	0.311
Residency area, Urban (%)	67	64	0.706	68	60	0.274
Ethnicity, Non-Indigenous (%)	71	68	0.727	61	58	0.752
Educations (grades of schooling in years, mean ± SE)	5.4 ± 0.12	5.5 ± 0.11	0.480	13.3 ± 0.4	12.1 ± 0.6	0.090
Education, adults (% six grades or higher)	NA	NA	NA	94	82	0.01

GDA: Guideline for nutritional amount. FOPWL: front-of-package warning label system. Sample size: GDA = 193, FOPWL = 162. SE: Standard error NA: not aplicable

P values obtained from t test and Chi square test

#### Distribution of outcomes

Descriptive statistics for all outcomes by label condition and phase are presented in Table 2. The comparison between FOPWL vs. GDA during phase 3 – after corresponding intervention labelling was depicted – shows that both, children and adults exposed to FOPWL improved significantly their objective understanding of products with excessive amounts of critical nutrients to GDA when comparing two products (p < 0.001). The purchase intention and the healthfulness perception of products with excessive amounts of critical nutrients decreased among children and adults exposed to FOPWL, compared to those exposed to GDA (p < 0.001). Similarly, when comparing two products, children and adults seeing FOPWL improved their purchase intention and healthfulness perception scores, compared to those exposed to GDA (p < 0.01).

**Table 2:** Distribution of outcomes in children and adults by label condition and phase, Guatemala, 2019

	Adults (n = 178)				
	Phase 1	Phase 3	Phase 1	Phase 3	p-value
Outcome ^**a**^	**GDA (n = 101)**		**FOPWL (n = 77)**		
Understanding of nutritional content indicator (1-100) (single product), mean ± SE	51.0 ± 1.1	62.1 ± 1.3	55.1 ± 1.2	72.6 ± 1.7	< 0.001
Understanding of nutritional content score (1-100) (comparison task), mean ± SE	19.9 ± 0.8	17.4 ± 0.8	20.7 ± 0.9	44.2 ± 2.3	< 0.001
Purchase intention indicator (1-100) (single product), mean ± SE	64.2 ± 1.7	60.9 ± 1.7	63.4 ± 2.0	38.0 ± 1.9	< 0.001
Purchase intention score (0–4) (comparison task), mean ± SE	1.2 ± 0.1	1.2 ± 0.1	1.4 ± 0.1	2.2 ± 0.1	< 0.001
Healthfulness Perception indicator (1-100) (single product), mean ± SE	51.4 ± 1.8	49.5 ± 1.0	48.3 ± 2.3	32.5 ± 1.9	< 0.001
Healthfulness Perception score (0–4) (comparison task), mean ± SE	1.3 ± 0.1	1.1 ± 0.5	1.6 ± 0.1	2.4 ± 0.1	< 0.001
	**Children (n = 177)**				
	**GDA (n = 92)**		**FOPWL (n = 85)**		
Understanding of nutritional content indicator (1-100) (single product), Mean ± SE	49.8 ± 1.0	55.0 ± 1.4	52.2 ± 1.2	59.2 ± 1.8	0.0625
Understanding of nutritional content score (1-100) (comparison task), Mean ± SE	24.6 ± 0.8	23.7 ± 0.7	25.6 ± 1.0	40.0 ± 2.2	< 0.001
Purchase intention indicator (1-100) (single product), mean ± SE	69.8 ± 2.1	61.0 ± 2.0	66.8 ± 1.9	44.0 ± 2.0	< 0.001
Purchase intention score (0–4) (comparison task), mean ± SE	1.4 ± 0.1	1.3 ± 0.1	1.4 ± 0.1	1.9 ± 0.1	< 0.001
Healthfulness perception indicator (1-100) (single product), mean ± SE	53.3 ± 2.1	53.0 ± 2.0	51.4 ± 2.0	38.7 ± 1.7	< 0.001
Healthfulness perception score (0–4) (comparison task), mean ± SE	1.8 ± 0.1	1.7 ± 0.1	1.7 ± 0.1	2.0 ± 0.1	0.003

P values obtained using a T test comparing FOPWL vs. GDA during phase 3 (exposure to intervention)

GDA: Guideline for Daily Amount system. FOPWL: front-of-package warning labeling system. SE: standard error

^**a**^**Understanding of nutritional content indicator (single product)**: Correct responses were scored 1, and the total number was obtained by adding the correct responses, converted into a 1-100 scale. **Understanding of nutritional content score (comparison task)**: Correct responses were scored 1, and the total number was obtained by adding the correct responses, converted into a 1-100 scale. **Purchase intention indicator (single product)** was estimated from a Likert Scale (1–7) and converted into 1-100 scale. **Purchase intention score (comparison task)**: The correct responses were given a score of 1 and the total score was obtained by adding the number of correct answers. **Healthfulness perception indicator (single product)**: was estimated from a Likert Scale (1–7) and converted into a 1-100 scale. **Healthfulness perception of Healthiness score (comparison task)**: The correct responses were given a score of 1 and the total score was obtained by adding the number of correct answers

### Effect of FOPWL on the nutritional content understanding, purchase intention and healthfulness perception compared to GDA

Table 3 presents the results of the DD regression models used to assess the effect of FOPWL on the understanding of nutritional content, purchase intention and healthfulness perception compared to GDA’s. Effect estimates from model 1 and 2 did not differ significantly. Therefore, we will focus on the results from model 2.

**Table 3:** Association of FOPWL with understanding nutritional content, purchase intention and healthfulness perception compared with GDA.

Outcome^a^	Pooled		Adults		Children	
	FOPWL vs. GDA		FOPWL vs. GDA		FOPWL vs. GDA	
Understanding of nutritional content indicator (single product), β (95%CI)						
Model 1^b^	3.7	(-0.1,7.5)	7.0*	(1.2,11.6)	1.8	(-3.5,7.2)
Model 2^c^	3.7	(-0.1,7.5)	6.4*	(1.2,11.6)	1.7	(-3.5,7.1)
Understanding of nutritional content score (comparison task), β (95%CI)						
Model 1	20.4***	(17.0,23.9)	25.9***	(21.1,30.7)	15.3***	(10.4,20.2)
Model 2	20.4***	(17.0,23.9)	25.9***	(21.1,30.7)	15.3***	(10.4,20.2)
Purchase intention indicator (single product), β (95%CI)						
Model 1	-18.0***	(-23.3,-12.8)	-22.0***	(-28.9,-15.0)	-14.0**	(-21.8,-6.2)
Model 2	-18.1***	(-23.3,-12.8)	-22.0***	(-28.9,-15.0)	-14.0**	(-21.9,-6.1)
Purchase intention score (comparison task), OR (95%CI)						
Model 1	4.5***	(2.9, 7.0)	5.7***	(2.3, 14.1)	3.7***	(2.0, 6.9)
Model 2	4.5***	(2.9, 7.0)	5.7***	(2.3, 14.4)	3.8***	(2.0, 7.2)
Healthfulness perception indicator (single product), β (95%CI)						
Model 1	-13.2***	(-18.4, -7.9)	-14.0***	(-21.1, -7.0)	-12.5**	(-20.3, -4.8)
Model 2	-13.2***	(-18.4, -7.9)	-14.0***	(-21.1, -7.0)	-12.5**	(-20.3, -4.7)
Healthfulness perception score (comparison task), OR (95%CI)						
Model 1	5.5***	(2.8, 10.8)	10.7***	(4.3, 26.7)	3.1**	(1.5, 6.5)
Model 2	5.6***	(2.8, 11.1)	10.8***	(4.3, 26.6)	3.2**	(1.5, 6.8)

*p ≤ 0.05, **p ≤ 0.01, ***p ≤ 0.001. Sample size: Adults = 178, Children = 177

GDA: Guideline for Daily Amount system. FOPWL: front-of-package warning labeling system. OR: Odds Ratio. 95%CI: 95% confidence intervals

^**a**^**Understanding of nutritional content indicator (single product)**: Correct responses were scored 1, and the total number was obtained by adding the correct responses, converted into a 1-100 scale. **Understanding of nutritional content score (comparison task)**: Correct responses were scored 1, and the total number was obtained by adding the correct responses, converted into a 1-100 scale. **Purchase intention indicator (single product)** was estimated from a Likert Scale (1–7) and converted into a 1-100 scale. **Purchase intention score (comparison task)**: The correct responses were given a score of 1 and the total score was obtained by adding the number of correct answers. **Healthfulness perception indicator (single product)**: was estimated from a Likert Scale (1–7) and converted into a 1-100 scale. **Healthfulness perception score (comparison task)**: The correct responses were given a score of 1 and the total score was obtained by adding the number of correct answers

^b^ Model 1 estimates are β coefficients or Odds ratios of the interaction term between label condition and phase of exposure controlling for label condition (FOPWL vs. GDA) and phase of exposure (Phase 3 vs. Phase 1) age and sex

^c^ Model 2: model 1 + residency (rural/urban), ethnicity (indigenous/nonindigenous) and education (6 grades or greater/less than 6 grades). In children education was used as a continuous variable (grades of schooling)

#### Understanding of nutritional content indicator (single product)

Model 2, the most adjusted one, showed that compared to GDA, FOPWL significantly improved the understanding about products’ nutritional content (single product task) in adults (β 6.4, 95%CI 1.2,11.6; p < 0.05). Similar results were found among populations with higher levels of education (β 10.9, 95%CI 5.4,16.4; p < 0.001) and those living in urban areas (β 7.4, 95%CI 2.4,12.5 p < 0.01) (see Additional file 3).

### Understanding of nutritional content score (comparison task)

During the product comparison task, FOPWL significantly improved the objective understanding of nutritional content by participants, when compared to those who were exposed to GDA (β 20.4, 95%CI 17.0,23.9; p < 0.001). Results were consistent in adults and children (β 25.9, 95%CI 21.1,30.7; p < 0.001 and β 15.3, 95%CI 10.4,20.2, p < 0.001 respectively) meaning that FOPWL increased the performance of interviewees to correctly identify products with excessive amounts of critical nutrients when comparing two products within the same food category. Significant interactions were found with area of residency and education in pooled analysis (p < 0.01), indicating that the effect of FOPWL was even greater in urban areas (β 24.9, 95%CI 20.4, 29.2; p < 0.001) compared to rural ones (β 12.7 95%CI 7.3,180; p < 0.01) (). Compared to GDA, FOPWL also improved the objective understanding of products’ nutritional content (comparison task) among participants with less than primary school level (β 13.9, 95% CI 9.4,18.5; p < 0.001), and the effect was even greater on those with higher level of education (β 30.1, 95% CI 25.0,35.2; p < 0.001) (see Additional file 3).

### Purchase intention indicator (single product)

Results from the most adjusted model also shows that individuals decreased the intention to purchase food products excessive in critical nutrients when seeing FOPWL, compared to those exposed to GDA when they evaluated single products (β -18.1, 95%CI -23.3, -12.8; p < 0.001). We found similar results in adults (β -22.0 95%CI -28.9, -15.0; p < 0.001) and children (β -14.0, 95%CI -21.9, -6.1; p < 0.01). In addition, the effect of FOPWL was similar among those living in rural areas and with less than 6 years of schooling. A significant interaction was found with education, where the reduction of purchase intention was even greater among those with higher level of education (β -23.2, 95%CI -30.5, -15.9; p < 0.001) (see Additional file 3).

### Purchase intention score (comparison task)

Compared to GDA, FOPWL provide a significant contribution to changing purchase intention score, by increasing the number of correct responses by four when comparing two products from the same category (OR 4.5, 95%CI 2.9, 7.0; p < 0.001). Similar results were found in children and adults (Table 3). In addition, FOPWL was effective to increase the number of correct responses among participants from rural areas (OR 4.9, 95%CI 3.7,6.5; p < 0.01) and among those with less than primary school (OR 3.7, 95%CI 2.2, 6.3 p < 0.01) compared to GDA. No interactions were found with area of residence and education. (see Additional file 3).

### Healthfulness perception indicator (single product)

Individuals exposed to FOPWL decreased by 13 points the perception of a food product as healthy during the single product task (β -13.2, 95%CI -18.4,-7.9; p < 0.001), when compared with GDA. In adults and in children these reductions were 14 points (β -14.0, 95%CI -21.1, -7.0; p < 0.001) and 12.5 points (OR -12.5, 95%CI -20.3, -4.8; p < 0.01) lower in the FOPWL group than in GDA’s, respectively. We also found similar results among those living in rural areas (β -12.0, 95%CI -20.4, -3.5; p < 0.001) and with less than 6 years of educations (β -11.4, 95%CI -18,7, -4.1; p < 0.01). (see Additional file 3).

### Healthfulness perception score (comparison task)

In the comparison task, FOPWL resulted in significantly higher odds for correctly identifying the healthier product more times (OR 5.6, 95%CI 2.8, 11.1; p < 0.001), compared with GDA. Results were consistent in children and adults (Table 3), and also in rural areas and among participants with less than 6 years of education (see Additional file 3). A significant interaction with education (p < 0.01) was found. Front-of-package warning labels increased by 13.6 times the odds in participants with more than primary education level (OR 13.6, 95% CI 5.1, 35.7; p < 0.001) and by 3 times in participants with less than primary education level (OR 3.1, 95%CI 1.3,7.2; p < 0.01) (see Additional file 3).

## Discussion

In this study, FOPWL significantly improved the participants’ objective understanding about the nutrient content of products with excessive amounts of critical nutrients when comparing pairs of products depicting the FOPWL system, compared with GDA. Similarly, adults and children exposed to FOPWL decreased their purchase intention and healthfulness perception over products excessive in total sugars, total fats, saturated fats, trans fats or sodium when they evaluated one product and pairs of products from the same category, compared with those exposed to GDA. These findings were independent from factors known to influence food choices such as age, sex, education, ethnicity and area of residence. The addition of such factors to the model did not change the estimates.

In children, we found no effect of the FOPWL on the understanding of nutrient content indicator, when evaluating single products, compared to GDA, however, when they compared two products from the same category, the FOWPL was an effective tool to identify excessive content of critical nutrients.

Our results on purchase intention and healthfulness perception were consistent among children and adults, including rural areas, and are in line with existing evidence carried out mostly in urban areas. For example in Morelos, Mexico, a randomized study showed that children (6-13y) increased their ability to select healthier choices compared with the traditional nutritional facts panel [35]. Another study in Montevideo, Uruguay showed that warnings labels discouraged children’s choices of unhealthy products compared to the traffic light system [42].

Similarly in adults, our results confirm what is known in previous research related with interpretative front-of-package systems in Canada, Mexico, Ecuador, Brazil and Uruguay, showing that industry sponsored GDAs are less impactful on food choices behaviors [31, 33, 43, 44]. Experimental studies in Brazil and Mexico further reported evidence suggesting that FOPWL lead consumers to perceive ultraprocessed food products as less healthy and provide better understanding of the nutritional content compared with similar food products labeled with the traffic light system [31] and GDA [32]. Randomized control trials using online shopping simulations in Mexico and Uruguay found that warning labels were an effective tool for guiding consumers towards healthier choices compared with GDAs [33, 34].

Overall, GDAs performed poorly in children and adults. Interpretative FOP labels such as warning labels might work through minimizing the effort and time – as they required less time during decision making –, and by raising the relevance of nutritional information with simple and salient icons [45, 46]. Additionally, FOPWL work by encoding information into working memory that facilitates identification of less healthier options [45–47].

The results of our study can be used to strengthen the current efforts made both in Guatemala (with the Law Bill 5504 “Promotion of Healthy Eating”), and at regional level with COMISCA´s proposal (Central American Technical Regulation). At regional level, our results show that it is the proposal of the Ministries of Health that should be implemented and not the industry-sponsored GDA’s.

Heterogeneity by education and area of residency was also found in our study. Overall, participants from urban areas and with higher levels of education (more than primary) understand even better when a product has excessive amounts of critical nutrients. They also have a better healthfulness perception of a product using the FOPWL. Heterogeneity across urban and rural areas, as well as education levels, on the understanding of nutritional content and healthfulness perception might be partly explained by the well-established association between education, nutritional status and dietary quality in Guatemala [48]. Education is a significant factor that can explain health inequalities and nutrition [49, 50], by affecting understanding of information, food choices and access to financial resources [49–52]. Nevertheless, the study demonstrated that FOPWL were efficacious even in populations with lower education levels. These results suggest that after the implementation of warning labels in Guatemala, communication campaigns could focus on more socially disadvantaged populations to further reduce disparities. The Chilean consumer awareness campaign issued by the Ministry of Health after the warning labels were implemented is a good example of such complementary initiatives, with messages like “Prefer foods with less labels” or “ No labels are even better ” [53, 54].

One of the strengths of the current study is that it is the first randomized experiment in Guatemala and Central America to assess the effect of FOPWL on food choices behaviors compared with GDA. In addition, this is the first study showing the efficacy of the FOPWL among adults and children from rural areas with an important proportion of indigenous population. Randomization might have minimized selection bias and confounding factors. Another strength is the use of previously validated instruments in the region that were tested and adapted for the Guatemalan context [31].

A limitation is that our results may not be widely applicable, since sample is not representative of overall Guatemalan population. Another potential limitation is that we used a conservative approach to calculate sample size which might have reduced power to detect differences. This is particular true for stratified analysis by type of participants, area of residency and education presented in this study. Studies with greater sample sizes are still needed in the region to confirmed these results. In addition, our sample has a greater proportion of females and of individuals that have attained more educational degrees than average women in Guatemala [49]. Nevertheless, our analyses were controlled for education and sex in adjusted models.

Another limitation is that participants were exposed to the FOPL icon of the group they were assigned to before they saw the products with such icon. That exposure may have increased their familiarity with the FOPL system and improved their skills. However, any potential improvement triggered by this familiarity would have affected both groups (GDA and FOWPL) equally, since they were exposed to same stimuli and the same order of questions. Therefore, the findings on the comparative advantage of one system over the other remain valid and unbiased.

## Conclusions

The results of the present study indicate that individuals exposed to FOPWL system improved their objective understanding about the presence of excessive amounts of critical nutrients related with NCDs in products. Exposure to FOPWL also decreased the purchase intention of products with excessive amounts of these nutrients and improved their misperception about these products’ healthfulness when evaluating single and pairs of products in children and adults, compared with GDA.

Conversely, GDA was inefficacious in improving consumers’ understanding about the nutrient content of products, their misperception about products healthfulness and their intention to purchase healthier options. Based on our findings, FOPWL ought to be adopted as part of a healthy food public policy in Guatemala. Its adoption as a food policy provides Guatemala, and Central American countries alike, the best opportunity to effectively meet the purpose of allowing consumers to easily identify products excessive in sugars, sodium, fats, saturated fats and trans fats, and take healthier food purchase decisions.

## Electronic supplementary material

Below is the link to the electronic supplementary material.

**Additional file 1.** Survey questions and translation into Spanish, Guatemala, 2019.

**Additional file 2.** Mock-ups of food products by label condition and nutritional information.

**Additional file 3.** The association of front-of-pack warning label system with understanding nutritional content, purchase intention and healthfulness perception compared with guidelines for daily amount in children and adults by area of residence and by level of education, Guatemala, 2019.

**Additional file 4.** Aims and Scope statement.

## Data Availability

The Institutional Ethics Committee (IEC) of the Institute of Nutrition of Central America and Panama (INCAP) has imposed restrictions to made de-identified data sets publicly available since data contains potentially sensitive information. This study was conducted in a small group of individuals from selected schools in urban and rural areas; including indigenous women and children that are considered vulnerable populations. Due to these privacy considerations imposed by INCAP’s IEC, the data are not publicly available, however data could be available upon request. Requests for access to the data may be made to the corresponding author and to the President of the INCAP Institutional Ethics Committee by researchers whose activities are reviewed by a Research Ethics Committee and who agree to sign an appropriate confidentiality agreement. Communication should be addressed to the President of INCAP IEC: Valentina Santacruz vsantacruz@incap.int.

## Abbreviations

FOPWL: Front-of-package warning labels
GDA: Guidelines for Daily Amount
HP: Healthfulness perception
PI: Purchase intention
UNC: Understanding of the nutrient content
NCDs: Non-communicable diseases
PAHO: Pan American Health Organization
WHO: World Health Organization
COMISCA: Council of Ministries of Health of Central America and Dominican Republic
SIECA: Central American Secretariat for Economic Integration
FOPL: front-of-package label
FAO: Food and Agriculture Organization
DD: difference-in-difference analysis
SE: Standard error
NA: Not applicable
CI: Confidence interval
