# Supplementary material for: Efficacy of front-of-pack warning label system versus guideline for daily amount on healthfulness perception, purchase intention and objective understanding of nutrient content of food products in Guatemala: a cross-over cluster randomized controlled experiment
Source: Arch Public Health. 2023 Jun 16;81:108. doi: 10.1186/s13690-023-01124-0 (PMC10273755; doi:10.1186/s13690-023-01124-0)
Supplement: Supplementary file 1 — Additional file 1. Survey questions and translation into Spanish, Guatemala, 2019. [file 13690_2023_1124_MOESM1_ESM.docx]

**Additional file 1.** Survey questions and translation into Spanish, Guatemala, 2019.

| **Indicator/score** | **Survey Questions (Spanish)** | **Response Scale (Spanish)** |
| --- | --- | --- |
| **Single product task-Participants see images of three products, one at a time** | | |
| Purchase intentions—Purchase  intentions | 1. Imagine que usted está comprando un [tipo de producto]. ¿Usted compraría este producto o un producto similar para usted y para su familia? | 7-point Likert scale:  (1) Seguramente no lo compraría, (2) no lo compraría, (3) es poco probable, (4) tal vez lo compraría, (5) es algo probable, (6) es muy probable, (7) seguramente sí lo compraría |
| Understanding of nutrient  content—Nutrient content score | 2. Hay profesionales que han establecido las cantidades de lo que una persona debe comer ¿Según su opinión este producto tiene cantidades elevadas de nutrientes de acuerdo a lo que dicen los profesionales? Le iré mencionando cada nutriente y usted me dirá si cree que está elevado de acuerdo a lo recomendado | (1) Grasa total, (2) Azúcar, (3) Sodio (sal), (4) Grasas trans (grasa no saludable), (5) grasa saturada (grasa no saludable), (6) Edulcorante (endulzante artificial), (7) ningún nutriente está por encima de los recomendado |
| Perceived  product healthfulness | Para usted, ¿este alimento es saludable? | 7-point Likert scale:  (1) Nada saludable, (2) Poco saludable, (3) Medianamente saludable, (4) Ni saludable ni no saludable, (5) Algo saludable, (6) saludable, (7) Muy saludable |
| **Product comparison task—Participants see images of two products at the same time** | | |
| Purchase intentions—Purchase intentions | Imagine que usted desea comprar un [tipo de producto]. ¿Usted compraría algún(os) de estos productos para usted o para su familia? Favor marcar 1 opción como respuesta | (1) Producto A, (2) Producto B, (3) Compraría los dos productos, (4) No compraría ninguno de los dos productos |
| Understanding of nutrient  content—Nutrient content score | ¿Cuál(es) de estos productos contienen mayores cantidades de grasas totales? | (1) Producto A contiene mayor cantidad, (2) Producto B contiene mayor cantidad, (3) Los dos productos tienen niveles altos, (4) Los dos productos tienen niveles bajos, (5) Ninguno de los dos, (6) No sabe/no responde |
|  | ¿Cuál(es) de estos productos contienen mayores cantidades de azúcares? | (1) Producto A contiene mayor cantidad, (2) Producto B contiene mayor cantidad, (3) Los dos productos tienen niveles altos, (4) Los dos productos tienen niveles bajos, (5) Ninguno de los dos, (6) No sabe/no responde |
|  | ¿Cuál(es) de estos productos contienen mayores cantidades de sodio? | (1) Producto A contiene mayor cantidad, (2) Producto B contiene mayor cantidad, (3) Los dos productos tienen niveles altos, (4) Los dos productos tienen niveles bajos, (5) Ninguno de los dos, (6) No sabe/no responde |
|  | ¿Cuál(es) de estos productos contienen mayores cantidades de grasas trans? | (1) Producto A contiene mayor cantidad, (2) Producto B contiene mayor cantidad, (3) Los dos productos tienen niveles altos, (4) Los dos productos tienen niveles bajos, (5) Ninguno de los dos, (6) No sabe/no responde |
|  | ¿Cuál(es) de estos productos contienen mayores cantidades de grasa saturada? | (1) Producto A contiene mayor cantidad, (2) Producto B contiene mayor cantidad, (3) Los dos productos tienen niveles altos, (4) Los dos productos tienen niveles bajos, (5) Ninguno de los dos, (6) No sabe/no responde |
|  | ¿Cuál(es) de estos productos contienen mayores cantidades de edulcorantes? | (1) Producto A contiene mayor cantidad, (2) Producto B contiene mayor cantidad, (3) Los dos productos tienen niveles altos, (4) Los dos productos tienen niveles bajos, (5) Ninguno de los dos, (6) No sabe/no responde |
| Product healthfulness—Product healthfulness score | ¿Puede indicarme qué producto considera más saludable? | (1) Producto A es más saludable, (2) Producto B es más saludable, (3) Los dos alimentos son saludables, (4) Ninguno de los dos es más saludable, (5) No sabe/no responde |
| **Label only task—Participants see the image of the label only** | | |
| Label understanding | Por favor, observe esta etiqueta y responda las preguntas a continuación.  Imaginemos que un producto tiene esta etiqueta nutricional. Según su opinión, ¿con qué frecuencia debe ser consumido un producto con esta etiqueta? | 7-point Likert scale:  (1) Nunca, (2) Muy raramente, (3) Raramente, (4) A veces, (5) Frecuentemente, (6) Muy Frecuentemente, (7) Siempre |
| Label understanding | Imaginemos que un producto tiene esta etiqueta nutricional. Según su opinión, ¿en qué cantidades debe ser consumido un producto con esta etiqueta? | 7-point Likert scale:  (1) Ninguna cantidad, (2) En muy pequeñas cantidades, (3) En pequeñas cantidades, (4) Ni en pequeñas ni grandes cantidades, (5) En medianas cantidades, (6) En grandes cantidades, (7) En muy grandes cantidades |
| Purchase intentions | ¿Si usted viera esta etiqueta en un producto que acostumbra comprar, que haría? | 7-point Likert scale:  (1) Ya no lo compraría, (2) Muy raramente lo compraría, (3) Raramente lo compraría, (4) A veces lo compraría, (5) Frecuentemente lo compraría, (6) Muy frecuentemente lo compraría (7) Lo seguiría comprando siempre |
| Label opinions | Ahora le vamos a presentar un empaque con una etiqueta. Le voy a leer unas oraciones y usted me dirá si está de acuerdo o no con lo que estoy diciendo  El etiquetado en el producto le llama la atención | 7-point Likert scale:  (1) Totalmente en desacuerdo, (2) Desacuerdo, (3) Desacuerdo parcialmente, (4) Ni de acuerdo o en desacuerdo, (5) De acuerdo parcialmente, (6) De acuerdo, (7) Totalmente de acuerdo |
| Label opinions | El etiquetado en el producto está visible |  |
