# Supplementary material for: Efficacy of front-of-pack warning label system versus guideline for daily amount on healthfulness perception, purchase intention and objective understanding of nutrient content of food products in Guatemala: a cross-over cluster randomized controlled experiment
Source: Arch Public Health. 2023 Jun 16;81:108. doi: 10.1186/s13690-023-01124-0 (PMC10273755; doi:10.1186/s13690-023-01124-0)
Supplement: Supplementary file 2 — Additional file 2. Mock-ups of food products by label condition and nutritional information. [file 13690_2023_1124_MOESM2_ESM.docx]

**Additional file 2.** Mock-ups of food products by label condition and nutritional information

| **Product, participants and task** | **Phase 1 (No Front-of-pack labelling o control condition)** | **Phase 3/**  **Octagon warning labels (FOPWL)** | **Phase 3**  **Guidelines for Daily Amounts (GDA)** |
| --- | --- | --- | --- |
| Yogurt 1  Adults urban areas  Single task  Comparison Task | 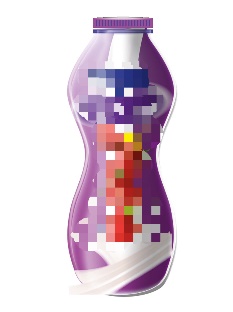 | 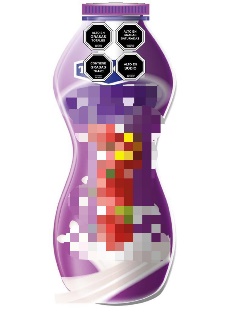High in total fat, high in saturated fat, contains trans fat, high in sodium | 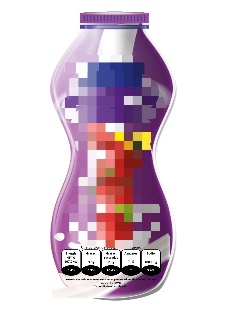  **A portion of 240 grams contains**  Energy 454 KJ 107.9 Kcal 5.4%  Fats 3.5g 5.3%  Saturated fat 2.3g 10.4%  Sugars 9.6g *  Sodium 108.1mg 5.4% |
| Yogurt 2  Adults urban areas  Comparison task | 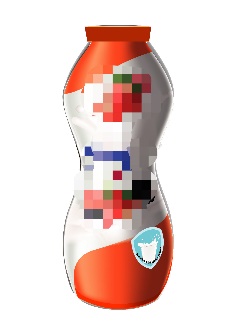 | 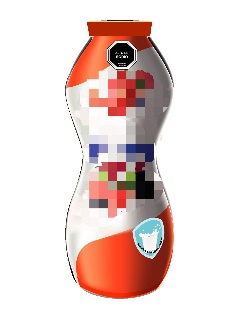High in sodium | 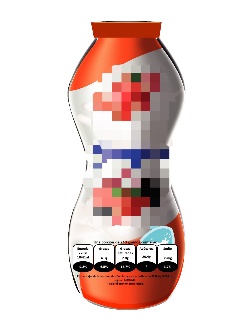  **A portion of 240 grams contains**  Energy 778KJ , 184 Kcal 9.2%  Fats 4.3g 6.5%  Saturated fat 2.8g 12.7%  Sugars 29.4g *  Sodium 75mg 3.7% |
| Cookie 1  Adults rural areas  Children  Single task  Comparison task | 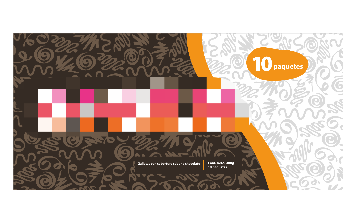 | 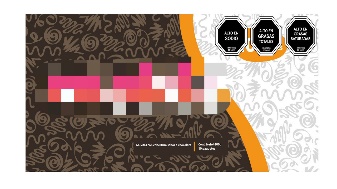High in sodium, high in total fat, high in saturated fat | 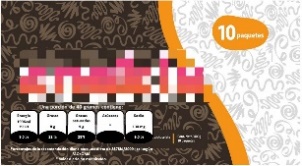  **A portion of 40 grams contains**  Energy 790 KJ 190 Kcal 9.5%  Fats 8g 12%  Saturated fat 5g 23%  Sugars *  Sodium 130mg 6.5% |
| Cookie 2  Adults rural areas  Children  Comparison task | 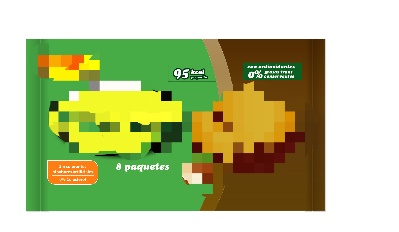 | 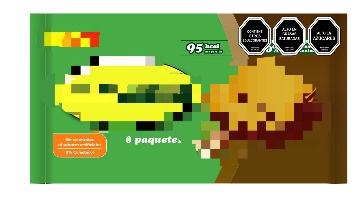  Contains artificial sweeteners, high in saturated fats, high in sugars | 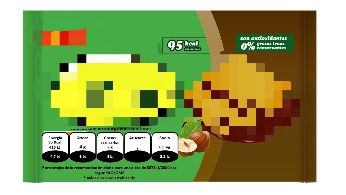  **A portion of 20.6 grams contains**  Energy 410 KJ 95 Kcal 4.7%  Fats 4g 6%  Saturated fats 2g 9%  Sugars *  Sodium 65mg 3.2% |
| Breakfast Cereal 1  Adults  Children  Single Task  Comparison task | 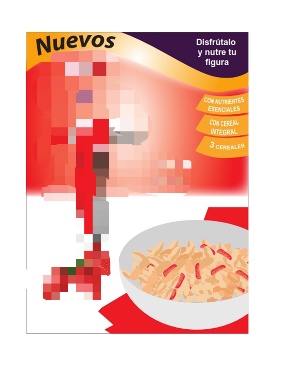 | 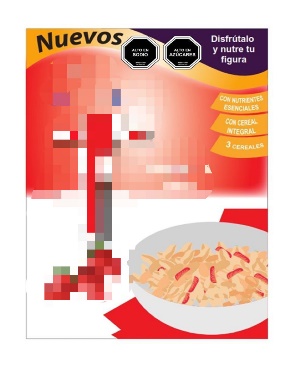High in sodium, high in sugars | 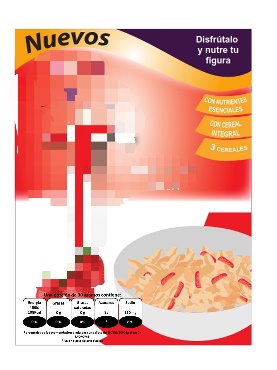**A portion of 30 grams contains**  Energy 459 KJ 108Kcal 0%  Fats 0g 0%  Saturated fats 0g 0%  Sugars 8g *  Sodium 150mg 8% |
| Breakfast Cereal 2  Adults  Children  Comparison task | 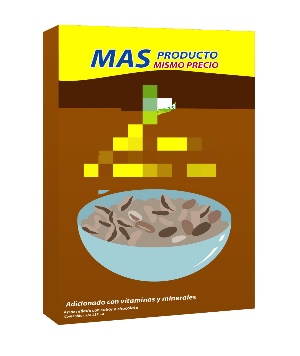 | 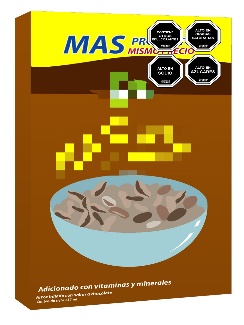  Contains artificial sweeteners, high in saturated fats, high in sodium, high in sugars | 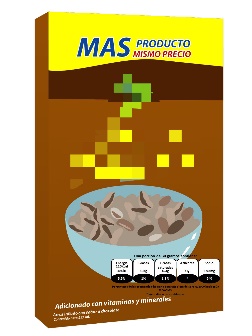  **A portion of 30 grams contains**  Energy 480KJ 110Kcal 5.5%  Fats 0.5g 1%  Saturated fats 0.3g 1.3%  Sugars 11g *  Sodium 180mg 9% |
| Soup 1  Adults  Comparison task | 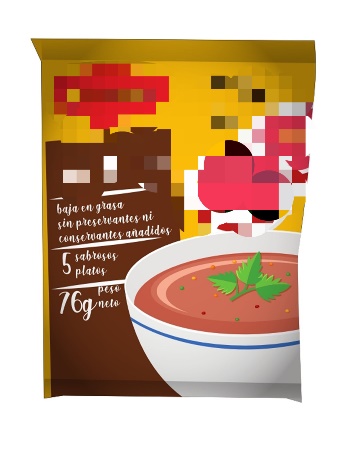 | High in sodium, high in total fats, high in saturated fats 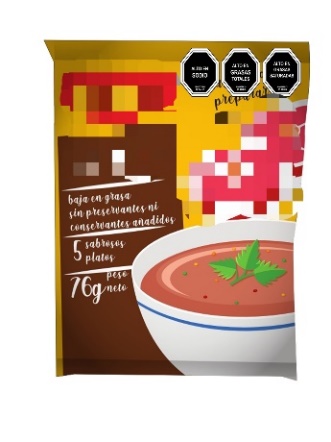 | 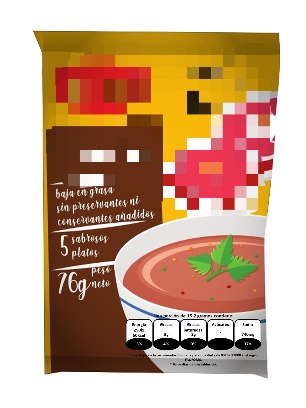**A portion of 15.2 grams contains**  Energy 250 KJ 60 Kcal 3%  Fats 3g 4%  Saturated fat 2g 9%  Sugars *  Sodium 740mg 37% |
| Soup 2  Adults  Comparison task | 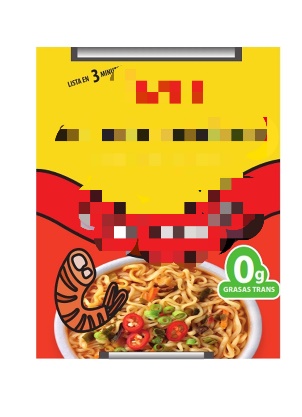 | 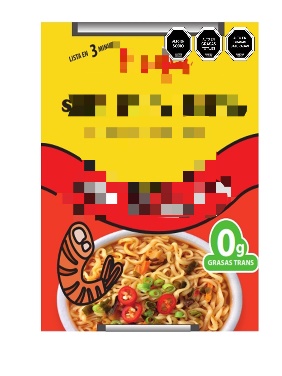High in sodium, high in total fats, high in saturated fats | 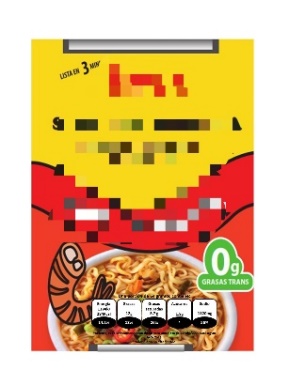**A portion of 64 grams contains**  Energy 1194 KJ 284 Kcal 14.2%  Fats 12g 18%  Saturated fat 5.7g 26%  Sugars 1.8g *  Sodium 1120mg 56% |
| Beverage 1  Adults  Single Task | 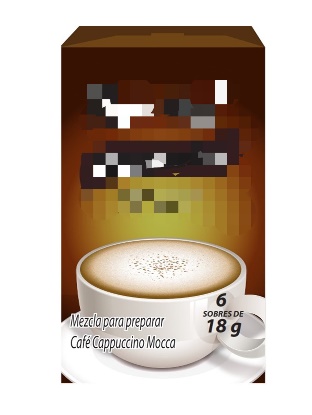 | 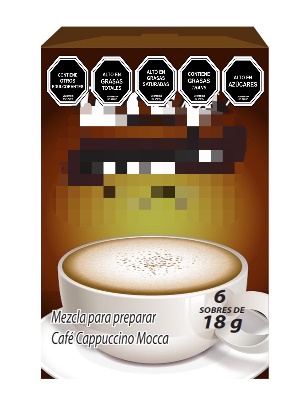Contains artificial sweeteners, high in total fats, high in saturated fats, contains trans fats, high in sodium | 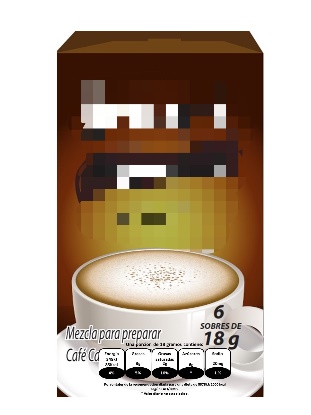**A portion of 18 grams contains**  Energy 348 KJ 83 Kcal 4%  Fats 3g 5%  Saturated fats 2g 10%  Sugars 6g *  Sodium 20mg 1% |
| Beverage 2  Children  Single task  Comparison task | 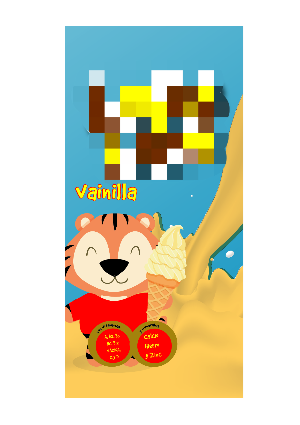 | 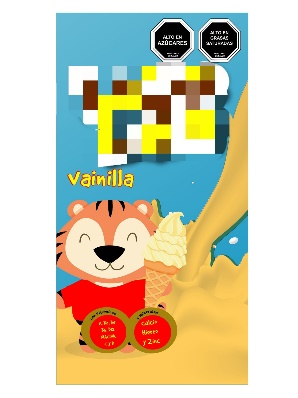  High in sugars, high in saturated fats | 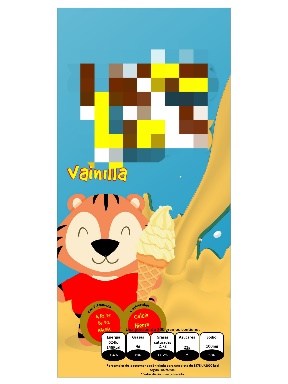  **A portion of 236 grams contains**  Energy 624KJ 148Kcal 7.4%  Fats 4g 6%  Saturated fats 2.7g 12.2%  Sugars 21g *  Sodium 100mg 5% |
| Beverage 3  Children  Comparison task | 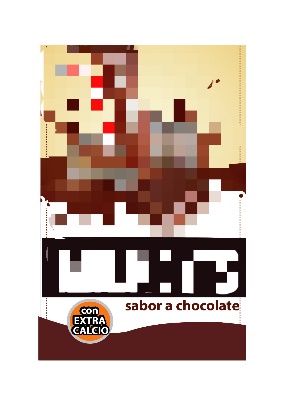 | 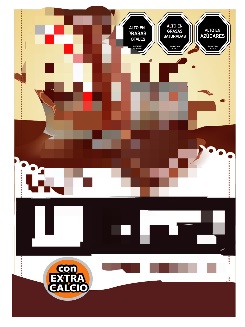  High in total fats, high in saturated fats, high in sugars | 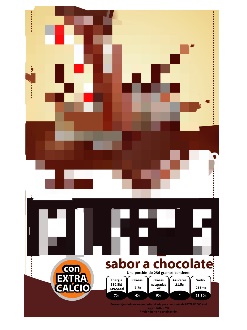  **A portion of 236 grams contains**  Energy 589.5KJ 139.5Kcal 7%  Fats 2.7g 4%  Saturated fats 2g 9%  Sugars 21.5g *  Sodium 266mg 13.3% |

All products with GDA presents this additional label: Percentages of the recommended daily intake for a diet of 8378Kj/2000Kcal according to FAO/WHO. * Daily value not established
