# Supplementary material for: Efficacy of front-of-pack warning label system versus guideline for daily amount on healthfulness perception, purchase intention and objective understanding of nutrient content of food products in Guatemala: a cross-over cluster randomized controlled experiment
Source: Arch Public Health. 2023 Jun 16;81:108. doi: 10.1186/s13690-023-01124-0 (PMC10273755; doi:10.1186/s13690-023-01124-0)
Supplement: Supplementary file 3 — Additional file 3. The association of front-of-pack warning label system with understanding nutritional content, purchase intention and healthfulness perception compared with guidelines for daily amount in children and adults by area of residence and by level of education, Guatemala, 2019. [file 13690_2023_1124_MOESM3_ESM.docx]

| **Outcome^a^** | **Urban^b^** | | | **Rural** | | **Primary^c^** | | **Less than primary** | |
| --- | --- | --- | --- | --- | --- | --- | --- | --- | --- |
| **Understanding of nutritional content** | FOPWL vs GDA | | | FOPWL vs GDA | | FOPWL vs GDA | | FOPWL vs GDA | |
| Single product, β (95%CI) | 7.4** | (2.4,12.5) | -1.92 | | (-7.2,3.3) | 10.9*** | (5.4,16.4) | 0.98 | (-4.8,5.1) |
| Comparison task, β (95%CI) | 24.9*** | (20.5,29.3) | 12.7** | | (7.3,18.0) | 30.1*** | (25.0, 35.2) | 13.9*** | (9.4, 18.5) |
| **Purchase Intention** |  | | |  | |  | |  | |
| Single Product, β (95% CI) | -19.4*** | (-26.2,-12.7) | -15.6*** | | (-23.9,-7.2) | -23.2*** | (-30.5,-15.9) | -13.9*** | (-21.4,-6.5) |
| Comparison task, OR (95% CI) | 4.4** | (2.3, 8.8) | 4.9*** | | (3.7, 6.5) | 6.4*** | (2.5,16.1) | 3.7*** | (2.2, 6.3) |
| **Perception of Healthfulness** |  | | |  | |  | |  | |
| Single product, β (95% CI) | -14.0*** | ( -20.7, -7.3) | -12.0*** | | ( -20.4, -3.5) | -16.3*** | (-23.8, -8.7) | -11.4** | (-18.7, -4.1) |
| Comparison task, OR (95% CI) | 4.4** | (1.5, 12.5) | 10.6*** | | (9.4, 11.8) | 13.6*** | (5.1,35.7) | 3.1** | (1.3, 7.2) |

**Additional File 3. The association of front-of-pack warning label system with understanding nutritional content, purchase intention and healthfulness perception compared with guidelines for daily amount in children and adults by area of residence and by level of education, Guatemala, 2019**

**p* ≤ 0.05, ***p* ≤ 0.01, ****p* ≤ 0.001. Sample size: Urban=232, Rural=124; Primary = 160, Less than primary ==196. ¿. Level of Education: Grades of schooling were measured in years and then dichotomized into 6 years or more (Primary), or less than 6 years (Less than Primary).

**^a^Understanding of nutritional content indicator (single product):** Correct responses were scored 1, and the total number was obtained by adding the correct responses, converted into a 1-100 scale**. Understanding of nutritional content score (comparison task):** Correct responses were scored 1, and the total number was obtained by adding the correct responses, converted into a 1-100 scale**. Purchase intention indicator (single product)** was estimated from a Likert Scale (1-7) and converted into a 1-100 scale. **Purchase intention score (comparison task):** The correct responses were given a score of 1 and the total score was obtained by adding the number of correct answers. **Healthfulness** **perception indicator (single product):** was estimated from a Likert Scale (1-7) and converted into 1-100 scale.  **Healthfulness perception score (comparison task):** The correct responses were given a score of 1 and the total score was obtained by adding the number of correct answers.

^b^ Area of residency: Estimates are β coefficients or Odds ratios of the interaction term between label condition and phase of exposure controlling for label condition (FOPWL vs GDA) and phase of exposure (Phase 3 vs Phase 1) + age, sex, level of education and ethnicity (indigenous/nonindigenous).

^c^ Level of Education: Estimates are β coefficients or Odds ratios of the interaction term between label condition and phase of exposure controlling for label condition (FOPWL vs GDA) and phase of exposure (Phase 3 vs Phase 1) + age, sex, area of residency and ethnicity (indigenous/nonindigenous).
