# Supplementary material for: Efficacy of front-of-pack warning label system versus guideline for daily amount on healthfulness perception, purchase intention and objective understanding of nutrient content of food products in Guatemala: a cross-over cluster randomized controlled experiment
Source: Arch Public Health. 2023 Jun 16;81:108. doi: 10.1186/s13690-023-01124-0 (PMC10273755; doi:10.1186/s13690-023-01124-0)
Supplement: Supplementary file 4 — Additional file 4. Aims and Scope statement. [file 13690_2023_1124_MOESM4_ESM.docx]

**Aims and Scope statement**

1. What is known?

Front-of-package (FOP) warning labels (FOPWL) that indicates when food products are excessive in total sugars, fats, saturated fats, trans fats or sodium and contains artificial sweeteners have been adopted in many Latin American countries aiming at reducing the consumption of unhealthy food and drink products. Given the growing evidence that FOPWL system is an effective health policy tool to improve consumers’ understanding, perception and purchase decisions, helping to tackle unhealthy diets and promote healthier food environments, it has been considered by the Central American Council of Ministries of Health and have also been considered in Bill 5504 in Guatemala (Healthy Promotion Eating Law). In contrast, the economy sector has proposed the Guideline Daily Amounts (GDA) FOP labeling in spite of the robust body of evidence that has demonstrated the ineffectiveness of that system. Previous research in Guatemala has found deficiencies in the declaration of nutrients related to NCDs in the form of nutrition facts tables. For example, food labeling technical regulations are not mandatory and an important proportion of processed and ultraprocessed products do not declare total sugars and trans fats. On the other hand, countries that have implement FOPWL have higher levels of urbanization and might have different responses to the warning labels compared with some Central American populations with a significant proportion of rural populations. To date, there is no evidence of the efficacy of both FOP systems in Central American populations where an important proportion of its habitants live in rural areas and have lower levels of education.

2. What does the study add?

This is the first randomized experiment conducted in Central America and in Guatemala, a low-middle country, with the highest rate of double burden of malnutrition in the western hemisphere with an important proportion of population living in rural areas. In this study we evaluated the efficacy of FOPWL versus the industry-sponsored GDA on products’ healthfulness perception, purchase intention and the objective understanding of nutrient content. Through a cross-over randomized experiment with children and adults, from rural and urban areas, we showed that FOWPL increases the correct understanding about the excessive amount of nutrients related with NCD´s (fats, sodium, sugar and artificial sweeteners) and promote healthier choices among participants. Results were similar among participants living in rural areas and with lower levels of education (less than 6 years of schooling), compared to GDA**.**

1. What are implications for clinical practice, public health and / or research?

A mandatory based-evidence front-of-pack food labelling in Central America is absent and currently national and regional authorities in Central America are discussing both front-of-package systems. Our results would immediate inform actions to Guatemalan government and central American authorities, showing that FOPWPL is more effective to address childhood obesity prevention and Non-Communicable Diseases in Central American populations. The GDA system was inefficacious in improving consumers’ understanding about the nutrient content of products, their misperception about products healthfulness and their intention to purchase healthier options. Based on this findings, FOPWL should to be adopted as part of a healthy food public policy in Guatemala and in Central America.
